# The work of managing multiple myeloma and its implications for treatment-related decision making: a qualitative study of patient and caregiver experiences

**DOI:** 10.1186/s12885-021-08527-8

**Published:** 2021-07-08

**Authors:** Cheryl Pritlove, Mobin Jassi, Bridget Burns, Arleigh McCurdy

**Affiliations:** 1 Applied Health Research Centre, St. Michael’s Hospital, 30 Bond Street, Toronto, ON M5B 1W8 Canada; 2 Faculty of Medicine, Queen’s University, 99 University Avenue, Kingston, ON K7L3P5 Canada; 3 Takeda Canada Inc, 24 Delhi Street, Guelph, ON N1E 4J6 Canada; 4 The Ottawa Hospital Research Institute, Box 704-501 Smyth Rd, Ottawa, ON K1H 8L6 Canada

**Keywords:** Multiple myeloma, Work, Patient, Caregiver, Decision making, Qualitative research

## Abstract

**Background:**

The evolving nature of multiple myeloma (MM) therapies, including the introduction of novel oral agents, has produced a shift in the delivery of care from hospital to home. Within this context, patients and their caregivers are becoming increasingly engaged in the work of illness management, however the exact nature of this work as well as the ways in which this work informs treatment preferences and decisions within this population has not been explored. This qualitative study sought to develop an in-depth understanding of patient and caregiver experiences with different MM treatments, the work necessitated by MM and treatment management, and the processes of patient/caregiver treatment-related decision making.

**Methods:**

Qualitative interviews were conducted with 16 MM patients and 8 caregivers. Interviews were coded for emergent themes and patterns and a constant comparative approach was used to identify important similarities and differences within and between interviews.

**Results:**

Patient and caregiver participants described four types of work, including the work of accruing and personalizing medical knowledge, illness-related work in the hospital, illness-related work in the home, and psychosocial and relational management. They illuminated the physical, psychological, social and relational toll of this work and traced a pathway through which work informed their treatment-related decisions, sometimes in ways that conflicted with their preferences for treatment.

**Conclusions:**

The work involved in managing MM, its treatment, and side-effects can inform the treatment decisions that patients and caregivers make. We must continue to find meaningful ways for patients and caregivers to discuss goals of care and treatment throughout the cancer trajectory, as well as support health care providers in the delivery of person-centred cancer care. With an increasing emphasis on the importance of shared decision making in MM, an improved understanding of the factors that frame patient’s and caregiver’s treatment decisions will be paramount to ensuring meaningful and high-quality patient-centered care.

## Background

Multiple myeloma (MM) is among the most common hematologic malignancies, characterized by periods of treatment, remission, and relapse along with significant morbidity and mortality [1]. In 2019, an estimated 3300 Canadians were diagnosed with MM, with approximately 1550 deaths within the same year [2]. While there is no cure for MM, advancements in the diagnosis, treatment, and management of this illness have contributed significantly to improved survival rates [3, 4]. Prognosis is expected to improve further with the introduction of new and novel proteasome inhibitors, immunomodulatory drugs, monoclonal antibodies, and epigenetic therapies [5, 6].

Historically, MM has been treated with conventional intravenous chemotherapy in hospital, however with the introduction of novel MM treatments in the last decade, including subcutaneous weekly bortezomib and the oral therapies Lenalidomide, Pomalidomide and Ixazomib, the standard of care for multiple myeloma patients is now overwhelmingly in the outpatient setting, with hospitalization required only in rare circumstances [1, 4, 6]. The result has been an overall shift in care from hospital to home, with patients and their caregivers becoming increasingly responsible for performing technical medical procedures, managing complex pharmaceutical regiments, as well as monitoring and managing treatment-related side-effects [6–9]. This work requires energy, action, time, and skill [10–12]; however, the extent and impact of this work on MM patients and their caregivers is not fully understood [8].

As MM is a heterogeneous illness with a multitude of treatment options available, patients are being increasingly encouraged to engage in treatment-related decisions [3, 13, 14]. As a result, patients and their caregivers must often learn about and assess multiple therapeutic options and, along with their health care provider, select the best treatment according to their unique needs and preferences [15]. There is evidence in the context of other cancer diagnoses that when multiple treatment options are available, personal factors and preferences, along with physician advice, play a key role in determining patients’ treatment decisions [16, 17]. While preferences data are available in the context of MM [18–21], little is known about how these preferences are formed and how they ultimately impact upon treatment-related decisions. With an increasing emphasis on the importance of shared decision making in MM [19, 22], an improved understanding of patient preferences and the factors underpinning those preferences will be paramount to ensuring meaningful and high-quality patient-centered care.

While the idea that illness and its management sometimes demand additional work on the part of patients and their caregivers is not new [9], the nature of work undertaken by MM patients and their caregivers as well as the ways in which the burden of work informs treatment preferences and decisions within this population has not been explored. Understanding the complex and nuanced factors that inform decision making among MM patients and their caregivers will be crucial to ensuring optimal and patient-centric decisions are made and that appropriate services and therapeutic modalities are provided. This qualitative study sought to develop an in-depth understanding of patient and caregiver experiences with different treatments, the work necessitated by MM and treatment management, and the processes of patient/caregiver treatment-related decision making.

## Methods

### Study design

We employed qualitative research methods, collecting data using in-depth, semi-structured qualitative interviews with MM patients and caregivers from across Canada. Given the national reach of the study, most interviews were conducted over the phone, requiring us to obtain verbal consent (in place of written informed consent). The study, including the consent process, was reviewed and approved by the St. Michael’s Hospital Research Ethics Board (REB#18–320). All eligible participants provided verbal informed consent prior to participating in the interview. Informed consent was recorded and transcribed.

#### Participant sampling and recruitment

We sought to recruit a total of 25 individuals, 15 multiple myeloma patients and 10 caregivers. Participants were recruited from the database of Myeloma Canada, a national patient advocacy organization. Myeloma Canada sent emails to their membership that included a study information letter and provided study information through their monthly newsletter. In addition, snowball sampling [23] techniques were used, whereby study participants shared information about the study with other MM patients and caregivers that they thought might be interested in participating. Those who were interested in the study were asked to reach out directly to the study coordinator, where they were given the opportunity to ask questions and were screened for eligibility. Given our aim to capture an in-depth understanding of patient and caregiver experiences with different multiple myeloma treatments, inclusion criteria required both patient and caregiver participants to have experience with two or more lines of MM treatment, experience with oral *and* IV/injectable therapies, and be at least 6 months post diagnosis. Participants were also required to be 18 years of age or over, English speaking, and have access to provincial health care coverage.

### Data collection procedures

Semi-structured interviews were conducted by telephone or in-person by an experienced qualitative researcher (CP) between June 2019 and February 2020. The interview guides were designed to capture patients’ and caregivers’ experiences of living with MM as well as perspectives and preferences regarding MM treatment. Topics included: the impact of MM on everyday life, experiences of managing MM and coping with treatment-related side-effects; experiences with and/or perspectives about MM treatment and treatment preferences; as well as financial costs associated with treatment. Demographic information was collected following the interview.

### Analysis

The interviews lasted between 65 and 120 min each. Interviews were digitally audio-recorded, transcribed verbatim, and quality checked to ensure accuracy [24]. Transcripts were reviewed in an iterative fashion that occurred simultaneously with data collection and were coded line-by-line to explore emergent themes and derive analytic concepts. Two researchers (CP, MJ) independently coded the data, and then held meetings to explore emerging codes and to develop, refine, and reach consensus on key codes and their definitions. A codebook [25] was generated through this collaborative process and applied to a second round of coding. Significant themes were identified as those that related directly to the project objectives, occurred consistently throughout individual interviews, and were discussed by several of the participants. Following this process, the transcripts were critically analyzed using a step-by-step approach to constant comparative analysis [26] to better understand differences and similarities within and between patient and caregiver groups. NVivo 11 data analysis software was used during the second-round of coding and for producing thematic reports of interview quotations. The analysis presented in this paper provides sample quotes to illustrate the broader themes identified.

## Results

### Participant characteristics

A total of 24 individuals (*n* = 16 MM patients and *n* = 8 caregivers) participated in the study, at which point it was determined that thematic saturation had been reached [27, 28]. Participants varied according to gender, age, living arrangements, education, employment status, and geographic location, as well as years living (or caring for someone) with MM. All caregiver participants were spouses of MM patients. Patient participants on the other hand described varying types of caregivers, including spouses, children, and friends. While some patient and caregiver dyads were interviewed (interviews conducted separately), most patients and caregivers were non-dyadic. Demographic information for these participants is summarized in Table 1.

**Table 1:** Demographic Characteristics for MM Patients and Caregivers

Characteristic	Patient (n)	Caregiver (n)
**Gender**		
Woman	10	7
Man	6	1
**Age** (Range (mean))	57–70 (65 years of age)	41–68 (62 years of age)
**Living Arrangements**		
Married	10	8
Alone	1	–
Divorced/separated	3	–
Single	2	–
**Family Structure**		
No children household:	14	7
Household with children:	2	1
**Education**		
High school	6	–
College	1	4
Undergraduate degree	7	3
Graduate degree(s)	2	1
**Employment status**		
Employed full time	2	4
Part time	1	–
Retired	10	3
Sick leave	3	1
**Private health Insurance**		
Yes	13	5
No	3	3
**Province**		
British Columbia (BC)	9	2
Alberta (AB)	2	–
Ontario (ON)	3	5
Newfoundland (NL)	1	–
Manitoba (MB)	1	1
**Distance to Cancer Centre**		
0–20 km	8	4
21-40 km	2	3
41-60 km	1	–
61-80 km	–	–
81-100 km	1	–
> 100 km	4	1
Years living with MM		
> 5 years	5	6
5–10 years	8	2
10–15 years	3	0

The participants in this study described in detail the physical toll of treatment and treatment-related side-effects (including pain, sleep disturbances, fatigue, neuropathy, and gastrointestinal issues), stressed the psychological and emotional impact of living with a chronic and incurable illness, and highlighted the social, relational, and practical disruptions experienced in everyday life. Participants did not endure these side-effects passively, but rather worked hard to adjust to life with MM and repair the many aspects of their lives that changed upon diagnosis. The participants in this study explained that the work of managing MM was diverse, time consuming, specialized, and required ongoing reprioritization. Participants described needing to rearrage work schedules and domestic tasks, aquire and interpret medical information, attend frequent medical appointments, perform complex and technical medical procedures (usually in the home), and locate necessary psychosocial and rehabilitative care and support. Participants identified four key types of work, including the work of *accruing and personalizing medical knowledge*, *illness-related work in the hospital, illness-related work in the home*, and *psychosocial and relational management*. In the following sections we describe these types of work, explore the social and relational contexts in which these tasks take place, and illuminate pathways through which patient and caregiver work affect treatment-related decisions.

### Accruing and personalizing medical knowledge

Participants explained that given the individualized nature of MM, the increasing amount of new and novel treatments available, and the lack of a ‘best’ medical therapy, they often found themselves in a position to select, along with their health care team, one or more treatments options. While these options provided comfort for many, particularly among those with many treatment options in the pipeline, it also necessitated intense periods of research to accrue needed knowledge as well as the skills necessary to interpret, assess, and apply mainstream medical knowledge to their particular circumstances. Below, a caregiver participant describes the work involved in becoming informed about MM, its trajectory, and the various treatment options available:

So, it was just trying to understand those [medical] terms and what they were trying to tell me was happening with them. I found that difficult. But again, I would just sit and research and figure it out. So, I was always doing seminars, like, you know, on Myeloma Canada, and the International Myeloma Foundation, they always have webinars. So I was signing up for every single one of those, (laugh) sitting there, at night, and trying to follow all those.

Participants emphasized the time invested into researching treatments and keeping up with new therapeutic developments, the assessment of which required a certain kind of expertise and skill. It was felt that not all MM patients and/or their caregivers had the time, energy, or possessed the savvy to negotiate this ever growing terrain of information, nor was it felt that all people had the capacity to translate this knowledge to their specific circumstances. As a patient participant explains:

It’s not easy trying to figure out all that stuff [different treatments]. I did a lot of reading, spoke to people from my support group to see if they had any experiences, talked to my doctors. And then you need to make the call, right. I mean I’ve got the time [retired] and my wife has a nursing background, so we could manage better than most people I think. So that is one potential gap, or crack maybe. Not everyone has those resources, I guess, to make informed decisions and advocate for what they want.

The patient and caregiver participants in this study described being confronted with complex decisions at various points in the course of their illness. Nearly all of the participants described being actively involved in treatment-related decisions, highlighting the importance of self-advocacy where care and treatment plans were concerned. However, the extent to which participants were involved in treatment decision making and their capacity to make informed decisions rested largely on their capacity to perform this work and to do so well.

### Illness-related work in the hospital

Diagnosis prompted immediate medical action, with participants describing an onslaught of medical tests, consults with specialists, and treatment appointments. Getting to treatment centres often required long travel and participants frequently described long waits for and during treatments. Attending these appointments was a time-consuming and demanding component of illness work that interrupted daily routines (e.g., employment, running errands, and household chores), thus competing with other important forms of work. It also interfered with activities they loved (e.g., reading a book, going for a walk or run, travel, gardening, playing with their grandchildren, and spending time with family and friends), with implications for overall well-being and quality of life. As the patient below explains:

So, it's hard, you know, when we're doing chemo every week, just the coming and going, it's about, it takes us about an hour and a half each way, to get to the hospital, because we have to take the ferry. There's a ferry line up. And, so you know, it pretty well kills the whole day… So, in a sense, you know, going for chemo, it really sort of interrupts a lot of our daily routine, you know, for a day or two there. And because, at the end of each cycle, we have to go in to see the chemo doctor. So we go in to see her the day before we do chemo, so that means two days in a row that we're off island. And you know, you don't get a lot done. So, and those days are tiring, and I end up sitting in the car a fair bit, and I don't get my walking done, which I try and do every day.

Living with an incurable illness and the looming threat of relapse was challenging for patients and caregivers alike. Indeed, participants spoke at length about the psychological and emotional impact of living with or caring for someone with MM as well as coping with the various effects of treatment. Managing physical symptoms and coping with the emotional challenges of living with MM, triggered what some referred to as a “patient identity”. This feeling of a lost self and the associated discomfort of living in a foreign or unrecognizable body were incredibly challenging for many of the participants. Indeed, many described active effort to return to a recognizable self, emphasizing their efforts in coping with these losses. As a patient participant explains:

I have to work at not getting down in the dumps. Cause I don't like that. Cause I'm normally a really happy person. So, I have to, I have to kind of work at, at not, um, obsessing about the multiple myeloma and reminding myself that I am so much more than my illness.

Frequent hospital visits tended to trigger this patient identity, a reminder of their illness and its terminality. This, even if temporarily, posed a symbolic threat to their efforts to live as normal a life as possible, with consequences for emotional well-being, amplifying the already challenging psychological toll of living with MM. Patients and caregivers described this as being emotionally difficult and emphasized efforts to cope with the anxiety and depression this posed in the days following hospital visits. As a caregiver participant explains:

Every time we went into the cancer clinic for when he was getting those shots once a week, it made him stressed out, because when you're at the cancer clinic, you're seeing all kinds of people with different kinds of cancer. But in his mind, they're just at different stages of death. So he was seeing all these people pretty darn sick.

The physical and emotional work associated with frequent hospital visits took a toll on both patients and their caregivers. While oral administration often meant additional work in the home (e.g. managing a pharmaceutical regimen), it was believed to be far less intensive and disruptive than that resulting from hospital visits. Participants described a preference for oral therapy because it permitted greater convenience and flexibility, reducing the need to rearrange work schedules and spend exhaustive amounts of time in hospital or travelling, as well as long waits for tests and treatments. Limiting exposure to the hospital and the patients therein also helped to limit reminders of their “patient identity” and the psychological toll associated with this position. In short, participants explained that oral administration minimized the overall burden of management work, and this route was often chosen when available. However, participants explained that they were happy to accept in-hospital treatments if they believed it to be more effective and/or have fewer side-effects than those delivered orally.

### Illness-related work in the home

While work occurred within and between hospitals/clinics, it was most prevalent in the home. With the introduction of complex and demanding medical tasks in the home, such as providing injections, managing pain and other side-effects, constantly sterilizing the home environment, and adhering to pharmaceutical regimens, care work became all-consuming for patients and their caregivers. This was particularly true during intense treatment times, including Stem Cell Transplant (SCT), with most participants describing either mandatory or strongly recommended outpatient recovery. Caregivers were often expected to shoulder the brunt of illness-related work in the home, particularly during treatment times when patients themselves were limited by physical impairments. When asked what the outpatient SCT was like from a caregiver’s perspective, the participant below explained:

Horrible. That was scary too…I had to take his temperature every hour, and write it down in their [hospitals] little book. Every time he drank something, I had to write it down, so it was keeping track of everything that he had and then, he had extra medication during that time. So, and it would change [every day]. So it was a lot more of a program…I did my best, but I'm not medically trained.

In discussing the round-the-clock provision of care paired with the complex care needs of their loved one, caregiver participants emphasized the medical skill (e.g. monitoring temperature and other vitals) required to perform this work. Indeed, few felt adequately prepared for the burden of work that fell on their shoulders. As a caregiver explains:

A lot of times, I kept saying out loud, 'I don't know what I'm doing. I'm not a nurse. And so, the responsibility that would be put on you is sort of like, 'I shouldn't be doing this. I'm not trained to do this.' So, it was a lot of, just questioning yourself, and your ability to do things.

The provision of this care was not only symbolically valuable – providing comfort, support and practical assistance for patients – but was also necessary, with such care often being required for the provision of certain treatments and medical procedures to take place. As a patient participant explains:

If you don't have a caregiver, someone who can be with you twenty four hours a day, someone who is willing to take you to and from the hospital every day, for a month or five weeks, or six weeks, it'll, you know, then you don't get the stem cell [transplant].

The availability of a caregiver, in turn became a key consideration in the treatment decisions available to a patient and the decisions they ultimately made.

A number of participants explained that the extent of medical-management work taken up by patients and their caregivers was somewhat invisible from a health systems perspective, and thus, insufficient care and supports required to cope with its impacts were provided. This was particularly true for caregivers, with participants emphasizing the lack of professional care and support available to this group.

Recognizing the largely unsupported burden of work taken up by caregivers in the home, some patient participants explained that despite a guiding preference for survival, that, in future, they would avoid treatments that required long-term and intensive caregiver support, such as a SCT, despite its promise for improved survival. In discussion of their experiences with an outpatient SCT, a caregiver participant explains that her husband, if confronted with the option of a second SCT, would either opt for an inpatient procedure or forego the transplant altogether:

For him, I know [he would choose inpatient] because it would take more stress off of me. Cause he was always worried about me. So I know for him, that would be [his choice], or he’d decide not do it.

This participant illuminates the ways in which the relegation of care to the home, and to the patient and caregiver therein, can be so intensive and challenging that it may ultimately inform treatment decisions in ways that can conflict with patient preferences. Indeed, several participants in this study explained that despite a clear preference for treatments that would provide the greatest chance for survival, this treatment option was not always pursued if it was believed that it would demand an unreasonable amount of work from them and/or their caregiver.

### Psychosocial and relational management

In describing the exhaustive and largely independent volume of work associated with medical-management, many patient and caregiver participants stressed the negative impact this work had on their psychosocial and relational well-being. For instance, as described above, caregivers assumed the bulk of illness-related work in the home, particularly during intense treatment times. The provision of this care in addition to their routine domestic and employment responsibilities often came at the expense of caregivers own health needs, with many emphasizing the ways in which the unconditionality of their care enhanced feelings of burnout and psychological distress. As a caregiver participant explains below:

I can't leave my job. I need a salary. I need the benefits for all these medications and whatnot. So, you just kind of 'do' and you just kind of go, and I probably, in hindsight, I probably should have sought a therapist for myself. And I didn't. I just, I relied on girlfriends to just, who were very supportive of me. You know, I probably should have done more for myself, but I didn't feel like I had the time, between work, kids and him [MM patient].

Patient participants spoke often about the impact of caregiver workload on their loved one’s quality of life. They expressed a sense of guilt for burdening their loved ones with worry, interrupting their social lives, and “derailing” plans they collectively made for the future (e.g. retirement plans, travel). As a patient participant explains below:

The things that we do together that we enjoy tend to be cut back a lot more, because we're spending more time doing the chores of life, the medical appointments, the resting, because I don't feel up to it.

Some treatments also made patients “edgier” and more “irritable”, causing tensions in their relationships. Despite working hard to manage the psychological and relational changes they endured as a result of MM treatment side-effects, participants often lacked the essential tools required to navigate these unfamiliar waters, rendering side-effects (including psychological impacts and disruptions to marital relationships) less manageable and more debilitating. As the caregiver below explains:

The dex really affected him [psychologically] and it affected our relationship, he was just so belligerent with me and he was never like that before, never. We wanted to get marriage counseling with someone who understands multiple myeloma and the drugs and the impact that those can have on someone, because I know that dex can make people really edgy, mean even. But we couldn’t find anything like that and the regular therapist we went to, she couldn’t handle us (laughs). His quality, both of our lives were really, the quality really went down and as hard as we tried to work through it, nothing was helping. So we decided to take dex out of the mix and were happy that our health care team listened…I had a few reservations that it would just take him longer to get better, if dex was taken out of the mix, because it is an effective drug...but he hated it. So it was a mutual decision to just come off of it.

Similar to the account above, many participants, particularly patients, described making treatment choices that would reduce their exposure to symptoms, minimize the impact of these symptoms on their or their loved ones, and/or minimize the burden of work involved in their management. This sometimes meant that the most effective treatment path was not always sought. While participants told accounts of health care providers who respected their wishes and altered therapy accordingly, these discussions rarely gave way to broader conversations with the health care team concerning why these decisions were being made. In turn, the work involved in managing side-effects remained invisible and opportunities to provide necessary supports were limited.

## Discussion

Participants in this study spoke in great detail about how MM and its treatment affected all aspects of their lives. They endured intense physical symptoms, profound changes in psychological and emotional well-being, as well as disruptions to their social, relational, and practical lives. Participants did not endure these side-effects passively, but rather described intense efforts to manage MM, cope with its treatment-related side-effects, and ultimately adjust, reconcile, and repair the many aspects of their lives that changed as a result of MM and its treatments. Participants emphasized the time, effort, and skill required to accrue and personalize medical information, coordinate and attend frequent medical appointments, manage varied and diverse symptoms and side-effects, continue with tasks of everyday living, and manage psychosocial and relational changes and challenges. Participants described making treatment choices that would reduce their exposure to symptoms, minimize the impact of these symptoms on their or their loved ones everyday lives, and/or minimize the burden of work involved in their management. This sometimes meant that the most effective treatment path was not always sought. This study thus adds to the existing MM literature by illuminating the all-consuming nature of work performed by patients and caregivers living with an incurable illness, describing the social and material resources required to perform these diverse tasks, and by highlighting the impact of patient/caregiver work on treatment-related decision making.

The intense and all-consuming nature of illness-management work captured in the current study has been discussed in previous cancer scholarship, confirming the complex nature of patient’s and caregiver’s work and the social and material resources needed to perform work tasks [7, 9, 12]. Our study extends this scholarship by exploring the work of patients and caregivers in the specific context of MM, an incurable illness which ebbs and flows between acute and chronic periods and for which medical and management work are not only ongoing, but constantly evolving. In discussing the burden of work and efforts to cope, the participants in this study stressed the vast and diverse impacts of multiple myeloma on their health and well-being and the subsequent need for an individualized and multimodal approach to care delivery. Like other studies with MM patients and caregivers [5, 7, 9, 14, 18], the participants in this study describe a need for psychosocial care that was described, at best, to be precariously available or unavailable. These findings reinforce decades of quantitative and qualitative evidence about the unmet care needs of cancer patients [29–31], but do so in a different health care milieu that increasingly pledges a commitment to person-centred and integrated care [32–34]. The lack of care integration and availability of psychosocial supports left many of the participants in this study feeling poorly equipped to adequately cope with side-effects and assume the burden of work demanded of them.

Participants’ workload, paired with individual capacity and resource accessibility, framed treatment decisions, with participants often opting for options that would minimize the overall burden of work and ultimately, the burden of illness. While most participants in this study had the necessary resources, support, and savvy to perform work-tasks, they also acknowledged that this was not always the case, and that many people living with this illness do not have the capacity or resources necessary to manage the varied, diverse, and demanding forms of work necessitated by MM and treatment-management. The inability to perform work tasks may limit one’s agency to make decisions that adequately reflect their preferences. This finding bears similarity to those from other studies that have documented the individualized and fluid nature of priority-setting and decision making in the context of MM [18] and assumes a sense of urgency as patients and their caregivers are being increasingly encouraged to engage in the decision making process to ensure their preferences are adequately reflected in the treatment paths pursued [3, 13, 14]. Understanding the complex and nuanced factors that inform decision making among MM patients and their caregivers is crucial to ensuring optimal and patient-centric decisions are made and that appropriate services and resources are provided to ease the burden of work.

This study confirms the invisibility of cancer-management work from a healthcare system perspective [12], and extends this scholarship by highlighting the ways in which this work can inform individuals’ treatment-related decisions. If the work of managing MM informs decision making, as suggested by the findings of this study, consideration of patient/caregiver work and the social and material resources it demands [9, 12] may need to factor more prominently in processes of shared decision making. While we recognize that oncology care teams are working within incredibly time constrained environments, which can place limits on what they are able/unable to accomplish in the delivery of care [34], we argue that inviting dialogue about such things as the emotional fallout associated with a MM diagnosis, experiences of managing care in the home, and the extent of social and/or care support available will likely enable providers to know more about, and better serve, patients. To this end, additional questions need to be raised in clinical encounters. For example, what kinds of support does the patient feel they have available to them within the home? Does the patient have the financial solvency to pay for a visiting nurse or do they have private benefits to cover these costs? How have social or spousal relationships changed post-diagnosis and how are patients and caregivers coping with these changes? And, does the patient and/or their caregiver appear to be overwhelmed or overtaxed? Questioning how people are coping, the work they are performing, and their access to resources may provide physicians with improved insight into patient/caregiver decision making pathways as well as enhance their understanding of the often invisible struggles that patients and caregivers are confronted with and the care supports they require as a result. Identifying conditions of struggle and working collaboratively with allied health professionals to ensure patients receive well-rounded and holistic care will not only support improved quality of life [12, 35–37], but may also help to enhance synergies between patients’ preferences for treatment and their treatment-related decisions [34].

There are limitations to this study that warrant consideration in review of the study findings. Firstly, all the patient participants had informal caregiving support. Given the practical and symbolic importance of caregivers as well as the burden of work/care they assume in the management of MM, this has important implications for the findings. It is possible that MM patients without such caregiver support may have different experiences and challenges than those captured in this study. Secondly, the participants in this study were recruited exclusively through Myeloma Canada, a national patient organization. While this provided access to patients and caregivers across Canada, the membership of this organization may be especially knowledgeable about and involved in the management of their myeloma. Thirdly, patients and caregivers shared their perceptions about their clinician’s beliefs, values and preferences with respect to decision making, however, the perspectives of the clinicians themselves are not represented. Additional research with clinicians to understand their preference for treatment and perceived role in the shared decision making process is warranted. Fourthly, the caregiver participants in our study were predominantly women and spouses of the person living with MM. Future research is needed to capture the experiences of a more demographically diverse sample of caregivers, including multiple genders, as well as those with varied relationships to the patient (e.g. friend, child, sibling, parent). Lastly, the findings should be interpreted in the context of a universal healthcare financing system. These findings may not necessarily align with the experiences of those who live in countries with private payer systems. Notwithstanding these limitations, the study provides further insight into topics relating to patient/caregiver work and decision making. Indeed, to the best of our knowledge, this study is the first to explore experiences of illness (and potentially competing forms of) work among MM patients and caregivers, providing additional understand into the underlying factors that inform patients’ and caregivers’ treatment-related decisions.

## Conclusions

Patients and caregivers living with MM were confronted with complex decisions at various points in their cancer journey relative to diagnosis, options for treatment, and how their disease and treatment choices may affect them physically, psychologically, emotionally, socially, relationally and practically. This study has helped to illuminate the interconnections between work, treatment preferences, and treatment-related decisions among MM patients and their caregivers. As such, it provides an important contribution to the literature by illuminating a complex, nuanced, and often invisible pathway to treatment-related decision making. The findings of this study suggest that the provision of care and supports to help manage the work necessitated by MM, its treatments, and its side-effects may ultimately impact upon the treatment decisions that patients and caregivers make. In turn, we must continue to find meaningful ways for patients and caregivers to discuss goals of care and treatment throughout the cancer trajectory, as well as support health care providers in the delivery of person-centred care. The use of decision aids that include considerations of this work and the burdens it can cause, may provide an important step in this direction.

## Data Availability

The datasets generated during the current study are available from the corresponding author on reasonable request.

## Abbreviations

MM: Multiple Myeloma
SCT: Stem Cell Transplant
